# Accurate and effective multidrug-resistant *Mycobacterium tuberculosis* detection method using gap-filling ligation coupled with high-resolution capillary electrophoresis-based single strand conformation polymorphism

**DOI:** 10.1038/srep46090

**Published:** 2017-04-19

**Authors:** Woong Choi, Jongseok Lee, Eunjin Cho, Gyoo Yeol Jung

**Affiliations:** 1 School of Interdisciplinary Bioscience and Bioengineering, Pohang University of Science and Technology, Pohang, Gyeongbuk 37673, Korea; 2 Section of Microbiology, International Tuberculosis Research Center, Masanhappogu, Changwon, Gyeongnam, 51755, Korea; 3 Department of Chemical Engineering, Pohang University of Science and Technology, Pohang, Gyeongbuk 37673, Korea

## Abstract

Tuberculosis (TB) has severely threatened public health via emerging multidrug-resistant (MDR) and extensively drug-resistant (XDR) *Mycobacterium tuberculosis* (MTB) strains. For effective TB treatment, rapid, accurate, and multiplex detection of drug resistance is extremely important. However, conventional methods for TB diagnosis are time consuming and have a limited effect on treatment. Nucleic acid-based molecular detection methods have been developed as an effective MDR/XDR-TB diagnosis technology. Among the nucleic acid-based methods, ligation-dependent methods are attractive as MDR/XDR-MTB detection technologies, but multiplex analysis is limited by the detection method. Although an electrophoresis-based method is considered for multiple target detection because it is free from the errors pertaining to hybridization-based systems, the procedure of multiplex analysis is quite complicated owing to the DNA size-based separation system. In this study, we report an accurate, rapid, and simple multiple MDR/XDR-MTB detection technology using gap-filling ligation reaction coupled with high-resolution capillary electrophoresis-based single-strand conformation polymorphism. Using this system, rapid and accurate MDR/XDR-MTB detection is feasible via similar length probes without the complicated step of probe design. We found that this method could accurately and effectively detect highly polymorphic regions in specific codons associated with drug resistance.

In recent years, tuberculosis (TB) has infected 9 million individuals and caused 1.5 million deaths worldwide^1^. In particular, extensively drug-resistant (XDR) TB, which cannot be cured by second-line antibiotics such as streptomycin (STR) and ciprofloxacin (CIP), was identified with multidrug-resistant TB (MDR-TB), which is resistant to first-line drugs such as rifampicin (RIF) and isoniazid (INH). Among the current cases of TB, the MDR type constitutes approximately 9.6%, and an increasing number of XDR strains are being reported every year^2^. Therefore, accurate and rapid detection of MDR/XDR-TB is essential for the diagnosis and treatment of TB.

Currently, drug susceptibility testing (DST) and Interferon-gamma (IFN-γ) release assay are widely used for TB diagnosis^3^,^4^,^5^. DST can accurately detect drug-resistant TB by the culturing of *Mycobacterium tuberculosis* (MTB) on a specific antibiotic medium. However, this method is time consuming. An alternative is the IFN-γ release assay, which allows more rapid detection than DST but exhibits false-positive and -negative results because of non-specific antigen–antibody interaction; furthermore, it cannot identify multidrug resistance.

MDR/XDR-TB is generated by various combinations of mutations spread in the nascent codons of the specific genes of MTB, defined as highly polymorphic regions^6^,^7^,^8^,^9^. These mutations leading to drug resistance are listed in Table 1. In particular, MDR/XDR-TB could occur if mutations were distributed in two or more drug-resistant genes. Furthermore, several highly polymorphic regions were very closely located in drug-resistant genes. Therefore, it is necessary to develop rapid, accurate, and multiplex nucleic acid-based multidrug TB detection technology.

Whole genome sequencing, line probe assay (LPA, Genotype^®^ MTBDR plus assay, HAIN Life Sciences, Nehren, Germany), reverse blot hybridization assay system (REBA), and multiplex allele specific PCR (MAS-PCR), and Xpert MTB/RIF assay (Cepheid, Sunnyvale, CA, USA) are representative commercial nucleic acid-based TB detection methods^10^,^11^,^12^,^13^,^14^,^15^,^16^,^17^,^18^,^19^. Whole genome sequencing method (WGS) hold the promise of affording genome-wide mutation detection on MTB genes. However, this method suffers from relatively high cost and time consuming process. As a hybridization-based method, line probe assay and REBA can detect multiple mutations of *rpoB, inhA*, and *katG* genes using Southern blot-coupled colorimetric reaction. Although this method can easily detect multiple targets leading to multidrug resistance, it provides false-negative or -positive signals generated by non-specific hybridization and thus requires elaborate hybridization condition control. In contrast, MAS-PCR can detect MDR or XDR MTB strains using multiple allele specific primers. Although this method can sensitively detect mutant genes involved multiple drug resistance, false-positive results are frequently occurred by non-specific hybridization of multiple primers. The Xpert MTB/RIF assay is based on real-time PCR using a molecular beacon probe. This method can sensitively detect RIF-resistant mutations generated in the *rpoB* gene using designed probes for highly polymorphic 81-bp core regions. However, the multiplexing power of this methodology is very low because of limitations such as dye availability for the detection of multiple mutations in MDR/XDR-MTB. Moreover, precise, simultaneous detection of closely located highly polymorphic regions is difficult using this assay because non-specific hybridization, such as the formation of probe dimer, could occur.

As alternative MDR/XDR-TB detection methods, ligation-dependent methods have been developed. These methods can accurately detect SNP sites to identify MDR/XDR-TB by minimizing non-specific hybridization because the ligation reaction using two specific probes per target occurs only if probes are specifically hybridized. The ligation-dependent methods currently available are the ligase detection reaction (LDR) and multiple ligation-dependent probe amplification (MLPA) assay^20^,^21^,^22^,^23^. LDR can amplify ligated products through the recurrence of denaturation and ligation reaction between probes and target templates, and MLPA is a method in which the ligation reaction occurs when probes are hybridized on targets; the ligated probes are then amplified using PCR. In case of the multiplex detection of closely located highly polymorphic regions, however, both assays were difficult because competitive hybridization could be generated by the similarity of the sequences between probes.

Ligated products generated by these methods are analyzed using bead array-based assay or an electrophoretic method. Bead array assay has an advantage in terms of the highly multiplexed detection of targets. However, because this detection system is based on the principle of DNA–DNA hybridization detection, non-specific hybridization is inevitable. Electrophoresis-based detection methods can be free from non-specific hybridization; therefore, they are not affected by errors because of hybridization-related problems. Nevertheless, they require length variation of final ligated products for detecting multiple targets. Therefore, custom design is difficult and the assay procedure is quite complicated because of the use of DNA size-based separation.

In this study, we report an accurate and rapid MDR/XDR-TB detection system using gap-filling ligation coupled with high-resolution capillary electrophoresis-based single-strand conformation polymorphism (GFL-CE-SSCP). This system comprises three simple steps: gap-filling ligation reaction, PCR amplification, and CE-SSCP separation, thereby providing a simplified custom assay design for MDR/XDR-TB detection. The gap-filling ligation reaction can accurately detect multiple highly polymorphic regions through two steps: the gap-filling reaction for hybridized probes designed to form a gap on the target using polymerase lacking exonuclease activity and nick ligation reaction^24^,^25^. Using these reactions, multiplex analysis for closely located highly polymorphic regions can not only eliminate competitive hybridization of probes but also effectively detect them. In addition, by introducing common primer sites in the designed probes, multiplex PCR can be applied using a single set of common primers. To detect multiple GFL products, CE-SSCP with highly enhanced resolution and separation was adopted. GFL products could be separated according to their unique folding structures depending on the sequence variations in the capillary filled with poly (ethyleneoxide)–poly (propyleneoxide)–poly (ethyleneoxide) (PEO-PPO-PEO) triblock copolymer, which we previously reported^26^,^27^,^28^. To establish this MDR/XDR-TB detection system, we selected highly polymorphic regions as targets for multiplex detection (Table 1). The results illustrate that our novel MDR/XDR-TB detection system provides an accurate and rapid TB diagnostic tool.

## Materials and Methods

### Probe design

Probes were designed to contain complementary sequences, both sides of which exclude highly polymorphic region, and by using (1) the Raw program to measure melting temperature and GC content of each probe, (2) ClustalW2 (http://www.ebi.ac.uk/Tools/msa/clustalw2/) to check the uniqueness of probe sequences, and (3) the Mfold server (http://mfold.rna.albany.edu/?q=mfold) to identify secondary structure formation of probes under reaction conditions. Left probe oligomers (LPOs) were designed to contain forward primer sequences (5′-GGGTTCCCTAAGGGTTGGA-3′) at the 5′-end. Designed right probe oligomers (RPOs) had the reverse primer sequences (5′-TCTAGATTGGATCTTGCTGGCAC-3′) at the 3′-end and were 5′-phosphorylated (Table 2). All designed probes were synthesized at Cosmogenetech (Seoul, Korea).

### Genomic DNA preparation of MTB

The strains having mutations on *rpoB, katG, inhA,* related to the first-line drug resistance, and *rrs, gyrA, gyrB,* related to the second-line drug resistance, were used in this study were selected to simultaneously detect MDR and XDR TB. Mutation sites of selected strains were showed in Table 2. MTBs were cultured in MB/BacT liquid culture (BioMérieux, Marcy l’Etoile, France) and on Löwenstein–Jensen (L-J) slants (ShinYang Chemicals, Seoul, Korea) at 37 °C for up to 8 weeks. gDNAs were extracted by boiling a suspension of mycobacteria scraped from L-J slants in 500 μl of distilled water; subsequently, this suspension was incubated at 99 °C for 30 min, and centrifuged at 13,000 × g for 5 min. After centrifugation, 5 μl of supernatant were used as template for GFL reaction^29^.

Genomic DNAs of sputum samples were extracted by handling sodium hydroxide and N-acetyle-L-cystein (NACL) on sputa. Processed sputum samples were subsequently inactivated for 10 min, and transferred to a microcentrifuge tube containing 200 μl of 0.1 mm glass beads. The tube was handled in a beads-beater (Biospec Products, Bartlesville, OK, USA) for 5 min and centrifuged at 13,000 × g for 5 min. The 5 μl of the supernatant were analyzed using GFL reaction as clinical samples. Genomic DNA of *M. tuberculosis* H37Rv (ATCC 27294) was used as standard in all GFL experiments.

### Multiplex GFL reaction

To detect highly polymorphic regions of MDRTB, multiplex gap-filling ligation reaction was performed in 50-μl volumes containing 20 nM probes, 1 U Vent exo^−^ polymerase, Taq DNA ligase (New England Biolabs, Ipswich, MA, USA), 1 × reaction buffer, 10 mM MgCl_2_, and 50 μM dNTP. Conditions for the gap-filling ligation reaction were pre-denaturation for 3 min at 94 °C, followed by 25 cycles of denaturation at 94 °C for 30 s and gap-filling extension and ligation at 65 °C for 1 min. This reaction was terminated by heat inactivation at 99 °C for 10 min.

Ligated products produced by this reaction were amplified using 5′-6-FAM-labeled forward primer (5′-FAM-GGGTTCCCTAAGGGTTGGA-3′) and reverse primer (5′-GTGCCAGCAAGATCCAATCTAGA-3′). PCR was performed in a volume of 20 μl containing 500 nM each primer, 2 μl of ligated products, and pfu PCR premix (Bioneer, Daejeon, Korea). The PCR thermocycling reactions comprised pre-amplification at 95 °C for 5 min, followed by 35 cycles of denaturation at 95 °C for 30 s, annealing at 60 °C for 30 s, and extension at 72 °C for 30 s, and then final extension at 72 °C for 7 min.

### CE-SSCP analysis

A total of 1 μl amplicon was mixed with 8.8 μl of deionized formamide (Applied Biosystems, Foster City, CA, USA) and 0.2 μl of GeneScan 500 ROX Size-standard (Applied Biosystems). The mixed samples were denatured at 95 °C for 5 min, followed by immediate cooling at 4 °C for 3 min. The cooled samples were injected into capillaries by applying a voltage of 15 kV for 5 s. Electrophoresis was performed for 1 h at 35 °C and a voltage of 15 kV. CE-SSCP analysis was conducted on the ABI3130xl instrument (Applied Biosystems) with 16 × 50 cm capillary arrays (Applied Biosystems). The capillary arrays were filled with 15 wt% PEO-PPO-PEO triblock copolymer (Pluronic F108; Sigma-Aldrich, St. Louis, MO, USA) dissolved in 0.7 × reaction buffer (Applied Biosystems).

## Results and Discussion

### Assay concept

**Figure 1:**
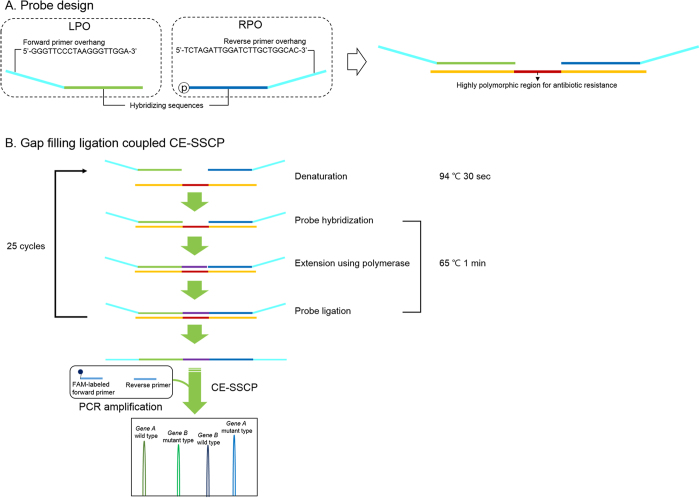
Illustration of the gap filling ligation coupled capillary electrophoresis-based single strand conformation polymorphism (GFL-CE-SSCP) assay. (**A**) GFL probe design and (**B**) strategy of the GFL-CE-SSCP assay.

MDR/XDR-TB detection technology described in this study was designed to accomplish an accurate and simple procedure for the sensitive detection of multiple mutations in highly polymorphic regions (Fig. 1). Probes were designed to sensitively detect multiple mutations present in specific codons; they have complementary sequences for both sides with a gap for a highly polymorphic region and common primer sites to amplify ligated probes and proceed to the GFL reaction using FAM-labeled forward and reverse primers. To analyze the multiple gap-filling ligated probes, signals from multiple probes have to be separated. In the conventional CE system, separation of these signals is allowed to use probes of different lengths. However, if probes become longer by length variation, accuracy for target detection is reduced due to non-specific hybridization caused by higher melting temperature or G/C contents, and formation of secondary structures. In our method, we adopted high-resolution CE-SSCP instead of a conventional CE system to avoid the complex probe design step by removing length variation of probes (Fig. 1A). In high-resolution CE-SSCP with PEO-PPO-PEO triblock copolymer developed previously, single-strand DNA with a unique conformation obtained by minor sequence variation can be separated even from single-strand DNA of similar lengths. Using wild-type genes as standard, MDR/XDR-TB mutants could be precisely detected as separated peaks (Fig. 1B).

### Detection of drug-resistant TB using GFL-CE-SSCP

Probes used for the GFL reaction were designed to detect mutations in *rpoB, katG*, and *inhA* genes generating resistance to first-line drugs such as RIF and INH, and *rrs, gyrA*, and *gyrB* genes causing tolerance to second-line drugs, including kanamycin (KAN) and fluoroquinolone. For multiplex analysis of mutations of each gene, folding structures of designed probes targeting mutations should have unique electrophoretic mobility in the CE separation system. Because the unique folding structure of ligated probes is associated with a difference in the free energy (ΔG) between denatured and folded states^30^, we considered that probes had distinctive ΔG values using the Mfold program. In addition, to prevent the non-specific hybridization of probes, sequence similarity was examined using the ClustalW2 program.

**Figure 2:**
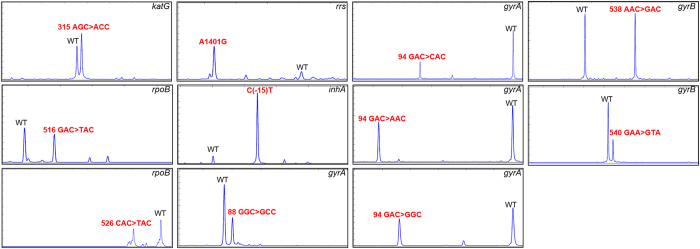
Multiplex analysis for mutation of each of the wild type and mutant genes using GFL-CE-SSCP. The electropherograms demonstrate that the wild type, defined as a standard, and mutants could be simultaneously detected. The x- and y-axes show migration time and relative fluorescence intensity (in arbitrary units), respectively.

To confirm the distinctive mobility of ligated products by GFL reaction, we performed a single GFL reaction for all targets. We found that peaks for each ligated product showed a specific migration time. Subsequently, multiplex GFL-CE-SSCP was conducted for detection of the wild type and mutants in highly polymorphic regions. As shown in Fig. 2, peaks for the wild type used as standard and mutant were clearly separated. In addition, relative migration times of the peaks for the wild type and mutants were observed to be reproducible in this multiplex assay; therefore, the wild type and mutants could be easily identified by the relative mobility in single and multiplex GFL-CE-SSCP analyses.

### Feasibility of multiplex GFL-CE-SSCP for MDR/XDR-TB detection

**Figure 3:**
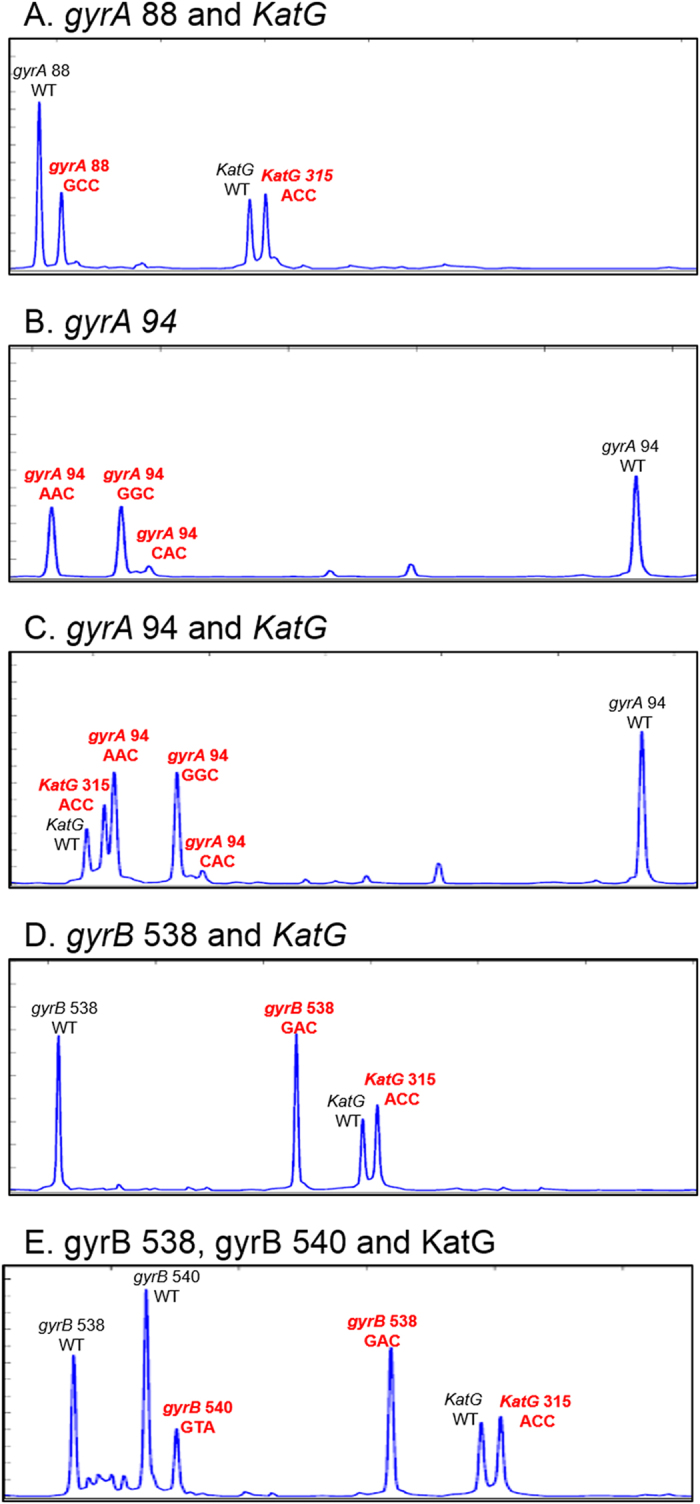
MDR- and XDR-MTB detection using multiplex GFL-CE-SSCP. These electropherograms prove the detection of MDR and XDR-MTB at the same time. (**A**) A codon 88 mutation of *gyrA* and a codon 315 mutation of *katG*. (**B**) Detection of a highly polymorphic region in codon 94 of *gyrA*. (**C**) A codon 94 mutation of *gyrA* and *katG* mutation. (**D**) A codon 538 mutation of *gyrB* and *katG* mutation. (**E**) Codon 538 and 540 mutations of *gyrB* and *katG* mutation. The x- and y-axes indicate migration time and relative fluorescence intensity (in arbitrary units), respectively.

**Figure 4:**
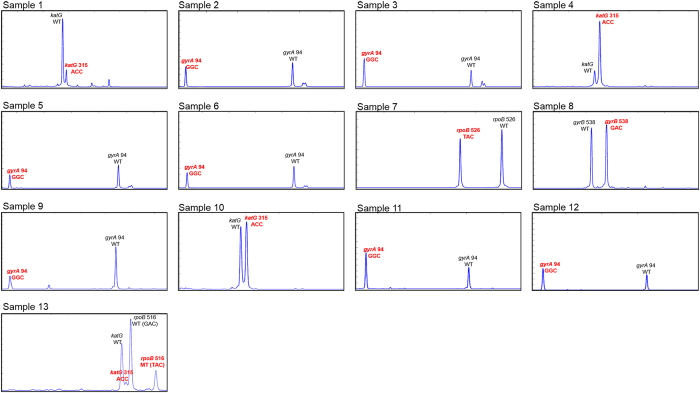
Multiplex analysis results on unknown samples. The 13 gDNA samples had various mutations causing MDR- or XDR-TB. For all electropherograms, the wild-type peak of each gene is used as a standard peak for mutant targets, and x- and y-axes indicate migration time and relative fluorescence intensity (in arbitrary units), respectively.

**Figure 5:**
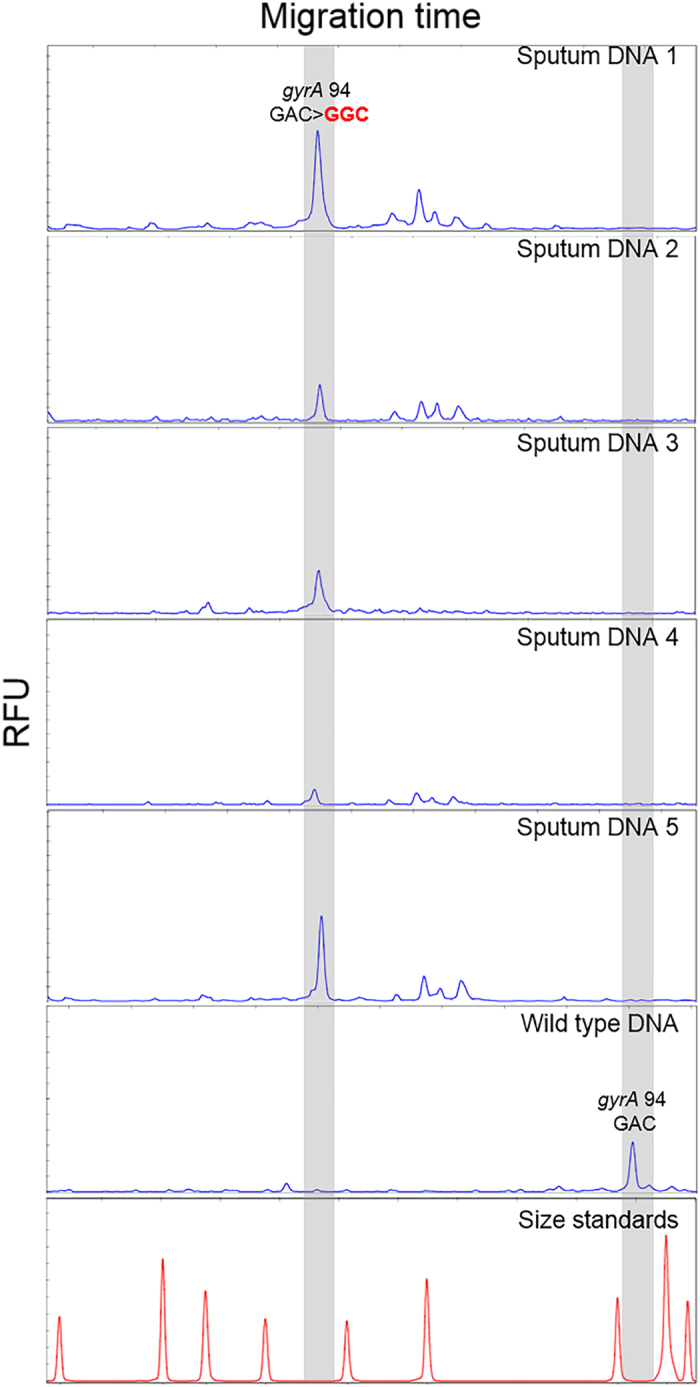
Detection of MTB isolated sputum samples using multiplex GFL-CE-SSCP. Migration of each sample peak was calibrated by size standards (bottom). All of the 5 clinical gDNA samples have mutations causing XDR-TB (codon 94 gyrA GAC > GGC). For all electropherograms, x- and y- axes show migartion time and relative fluorescence intensity (in arbitrary units), repectively.

For accurate TB diagnosis, multiple detection of drug-resistant genes is important. To perform MDR/XDR-TB detection, multiplex GFL-CE-SSCP was conducted for mixed TB DNAs containing mutations in each highly polymorphic region. As a result of the multiplex GFL-CE-SSCP analysis shown in Fig. 3, all highly polymorphic regions were separated by multiplex GFL-CE-SSCP as follows: mutations in codon 88 of *gyrA* (GGC > TGC) and codon 315 of *katG* (AGC > ACC), mutations in codon 94 of *gyrA* (GAC > AAC, GGC, CAC) and *katG*, mutations in codon 538 of *gyrB* (AAC > GAC) and *katG*, and mutations in codons 538 and 540 (GAA > GTA) of *gyrB* and *katG*. Therefore, we confirmed that MDR- and XDR-MTB were accurately detected using our GFL-CE-SSCP analysis at the same time. To confirm that our assay could be used for the detection of mutations in unknown and clinical samples, we analyzed 13 unknowns and 5 clinical samples isolated sputum. As shown in Fig. 4, the mutations caused in DNA of 13 unknown samples were multiply detected, and Fig. 5 indicates that the mutation in codon 94 of gyrA (GAC > GGC) on 5 clinical samples were accurately detected; this further coincided with the results of sequencing analysis. These results demonstrated that our assay can detect multiple highly polymorphic regions and suffers free from non-specific hybridization which can occur in hybridization-based methods. In addition, analysis time for MDR/XDR-MTB is faster than whole genome sequencing methods; total analysis time for multiple MDR/XDR MTB detection is about 5 h. Therefore, we proved that MDR/XDR-MTB were accurately detected and that rapid and multiplex detection was possible using our GFL-CE-SSCP assay.

## Conclusions

In this paper, we have described an accurate, rapid, and multiplex MDR/XDR-TB detection assay based on GFL coupled with a high-resolution CE-SSCP system. In our MDR/XDR-TB detection method, because gap-filling reactions were performed for highly polymorphic regions, false-positive or -negative results could be minimized by non-specific hybridization for target detection. Because of its customized design and simplified three-step process, i.e., gap-filling ligation, PCR, and CE-SSCP, and the entire reaction time for TB detection being about 5 h, the robust MDR/XDR-TB detection assay depicted here is an attractive method for the accurate diagnosis of TB.

## Additional Information

**How to cite this article:** Choi, W. *et al*. Accurate and effective multidrug-resistant *Mycobacterium tuberculosis* detection method using gap-filling ligation coupled with high-resolution capillary electrophoresis-based single strand conformation polymorphism. *Sci. Rep.* **7**, 46090; doi: 10.1038/srep46090 (2017).

**Publisher's note:** Springer Nature remains neutral with regard to jurisdictional claims in published maps and institutional affiliations.

## Figures and Tables

**Table 1:** Genetic mutations associated with multidrug-resistant tuberculosis.

Drugs	Drug targets	Drug resistance-related gene	Function of genes
Rifampicin	RNA synthesis	*rpoB*	DNA-dependent RNA polymerase
Isoniazid	Mycolic acid	*katG*	Catalase/peroxidase
	Biosynthesis	*inhA*	Fatty acid biosynthesis
Kanamycin	Protein synthesis	*rrs*	16S rRNA
Ciprofloxacin	DNA synthesis	*gyrA*	DNA gyrase subunits A and B
		*gyrB*	

**Table 2:** LPO and RPO sequences for highly polymorphic region detection by multiplex GFL.

Gene	Mutation site	LPO sequence (5′–3′)	RPO sequence (5′–3′)^a^
*rpoB*	516^b^ (GAC > TAC)	GGGTTCCCTAAGGGTTGGAGTCAACCCCGACAGCGGGTTGTTCTG	CATGAATTGGCTCAGCTGGCTGGTGCCTCTAGATTGGATCTTGCTGGCAC
	526^b^ (CAC > TAC)	GGGTTCCCTAAGGGTTGGAGACCAGAACAACCCGCTGTCGGGGTTGACC	CGACTGTCGGCGCTGGGGCCCTCTAGATTGGATCTTGCTGGCAC
*katG*	315 (AGC > ACC)	GGGTTCCCTAAGGGTTGGACGGGGTGTTCGTCCATACGACCTCGATGCC	GGTGATCGCGTCCTTACCGGTTCCGGTGCTCTAGATTGGATCTTGCTGGCAC
*inhA*	−15^c^ (C > T)	GGGTTCCCTAAGGGTTGGAGAAACGCGATCGACGAGTCGGTGAT	TCCGCTAACCAGAATCCGTTTGCCTCTAGATTGGATCTTGCTGGCAC
*rrs*	1401nt (A > G)	GGGTTCCCTAAGGGTTGGACGTCATGAAAGTCGGTAACACCCGAAGCCAGTG	GACGGGCGGTGTGTACAAGGCCCGTCTAGATTGGATCTTGCTGGCAC
*gyrA*	88 (GGC > GCC)	GGGTTCCCTAAGGGTTGGACACCAGGCTGTCGTAGATCGACGCGTC	GTGCGGGTGGTAGTTGCCCATGGTCTCTCTAGATTGGATCTTGCTGGCAC
	94 (GAC > AAC) 94(GAC > CAC) 94 (GAC > GGC)	GGGTTCCCTAAGGGTTGGAAGGGCTGGGCCATGCGCACCAGGCT	GTAGATCGACGCGTCGCCGTGCGGGTGGTAGTTTCTAGATTGGATCTTGCTGGCAC
gyr*B*	538 (AAC > GAC)	GGGTTCCCTAAGGGTTGGACGCCGTGATGATCGCCTGAACTTCGGT	CTTTAGCACCCGGTCGATGCGCGCTTTCTCTAGATTGGATCTTGCTGGCAC
	540 (GAA > GTA)	GGGTTCCCTAAGGGTTGGAGCCCAGCGCCGTGATGATCGCCTGAAC	GGTGTTCTTTAGCACCCGGTCGATGCGCGCTTTTCTAGATTGGATCTTGCTGGCAC

^a^All right probe oligos (RPO) are phosphorylated at the 5′-end.

^b^The number indicates the codon site.

^c^−15 means promoter region sites.
